# The anti‐migration effect of partially covered self‐expandable metal stents for unresectable malignant distal biliary obstruction: A multicenter comparative study

**DOI:** 10.1002/deo2.70100

**Published:** 2025-03-20

**Authors:** Shinya Kohashi, Arata Sakai, Keisuke Furumatsu, Takeshi Ezaki, Takao Iemoto, Takeshi Tanaka, Masahiro Tsujimae, Takashi Kobayashi, Atsuhiro Masuda, Yuzo Kodama

**Affiliations:** ^1^ Department of Internal Medicine Division of Gastroenterology Kobe University Graduate School of Medicine Hyogo Japan; ^2^ Department of Gastroenterology Akashi Medical Center Hyogo Japan; ^3^ Department of Gastroenterology Osaka Saiseikai Nakatsu Hospital Osaka Japan; ^4^ Department of Gastroenterology Kobe Medical Center Hyogo Japan; ^5^ Department of Gastroenterology Kita‐harima Medical Center Hyogo Japan

**Keywords:** bile duct neoplasms, endoscopic retrograde cholangiopancreatography, obstructive jaundice, pancreatic neoplasms, self‐expandable metallic stents

## Abstract

**Objectives:**

Covered self‐expandable metal stents are commonly used for unresectable malignant distal biliary obstruction. Partially covered self‐expandable metal stents have uncovered sections at both ends; however, their anti‐migration effect remains unclear. The objective of this study was to evaluate that effect by comparing such stents with fully covered self‐expandable metal stents for patients with unresectable malignant distal biliary obstruction.

**Methods:**

This was a multicenter, retrospective comparative study of partially covered stents with fully covered stents for unresectable malignant distal biliary obstruction. Stent migration, recurrent biliary obstruction, and the time to recurrent biliary obstruction were compared between them.

**Results:**

Thirty‐nine patients with partially covered stents were included and compared with 42 patients with fully covered stents. The partially covered group had a significantly lower stent migration rate (3% vs. 36%; *p *< 0.001). The recurrent biliary obstruction rate was significantly lower in the partially covered group (21% vs. 43%; *p *= 0.036). The non‐recurrent biliary obstruction rate at 6 months was 90% and 68% in the partially and fully covered groups, respectively. The time to recurrent biliary obstruction was significantly longer in the partially covered group (Gray's test, *p *= 0.016). Only partially covered stent placement was significantly associated with a lower risk of stent migration (subdistribution hazard ratio = 0.077; 95% confidence interval = 0.01–0.60; *p *= 0.014) in the multivariable analysis.

**Conclusions:**

The anti‐migration effect of partially covered self‐expandable metal stents was associated with a reduced recurrence of biliary obstruction and prolonged time to such obstruction.

## INTRODUCTION

Endoscopic transpapillary biliary drainage for patients with unresectable pancreatic or biliary cancer can relieve obstructive jaundice, improve quality of life, and allow continuation of the chemotherapy regimen. Hence, it is a standard procedure for the palliative management of such patients.

Self‐expandable metal stents (SEMSs) are commonly used for unresectable malignant distal biliary obstruction (MDBO) because they have a longer patency than plastic stents.^1^ SEMSs are divided into covered SEMSs (CSEMSs) and uncovered SEMSs (UCSEMSs). A meta‐analysis revealed that CSEMSs are superior to UCSEMSs in preventing recurrent biliary obstruction (RBO) in patients with MDBO, especially those caused by pancreatic cancer but have a significantly higher incidence of cholecystitis than UCSEMSs.^2^ Three randomized controlled trials from Japan revealed that CSEMSs have a longer patency than UCSEMSs,^3^, ^4^, ^5^ and Japanese clinical practice guidelines for pancreatic and biliary tract cancer recommend CSEMSs for patients with MDBO.^6^, ^7^

CSEMSs can be divided into fully CSEMSs (FCSEMSs) and partially CSEMSs (PCSEMSs). FCSEMSs are completely covered by a thin membrane, which prevents tumor ingrowth and simplifies their removal because they are not embedded in the bile ducts. In contrast, PCSEMSs have uncovered sections at both ends to reduce the risk of stent migration. To our knowledge, few studies have been conducted to compare FCSMESs and PCSEMSs, and those resulted in different conclusions regarding their anti‐migration effect.^8^, ^9^ Therefore, the effect of the uncovered sections of PCSEMSs on stent migration remains unclear.

A previous multicenter prospective study on the performance of FCSEMSs in unresectable MDBO revealed a high stent migration rate (36%).^10^ Therefore, we designed a retrospective study to compare the clinical outcome of PCSEMSs with that of the FCSEMSs in that study. The aim of this study was to evaluate the anti‐migration effect of PCSEMSs in comparison with FCSEMSs in patients with unresectable MDBO.

## METHODS

### Study design and patients

**FIGURE 1:**
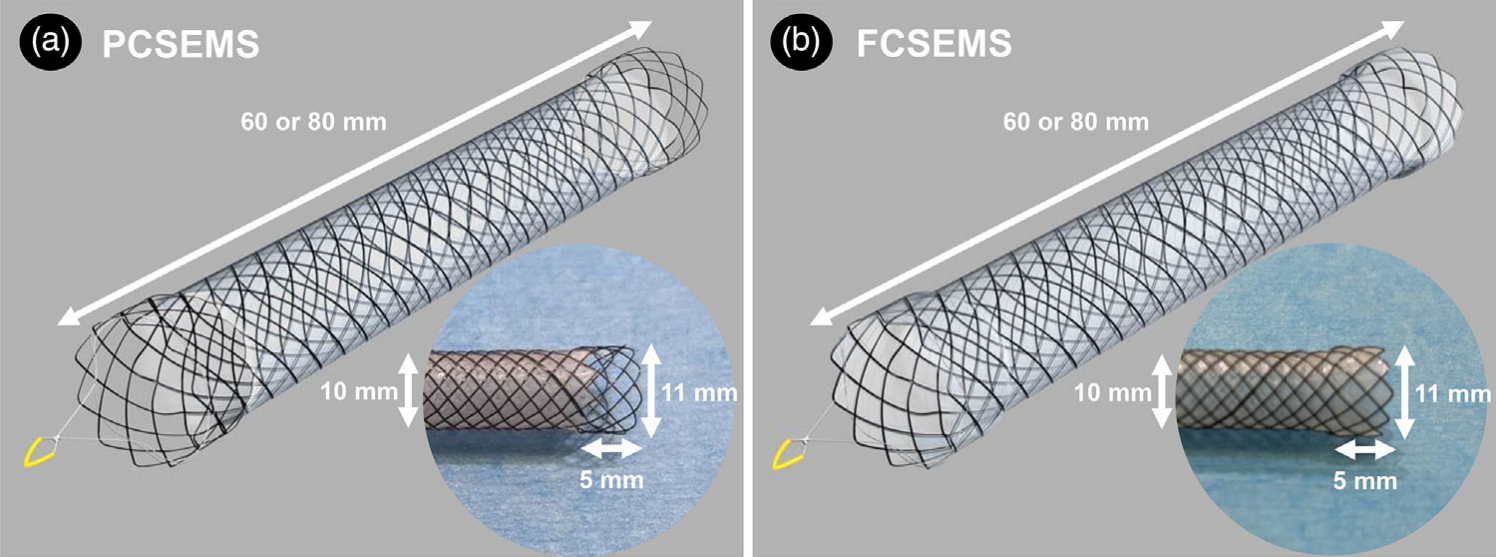
The PCSEMS (a) and FCSEMS (b) used in this study (Evolution Biliary Stent, Cook Ireland Ltd.) had a nitinol‐based braided cross‐wire structure with a platinum core and proximal and distal flares to reduce stent migration. The stent outer diameter was 10 mm, the flare outer diameter was 11 mm, the flare length was 5 mm and the stent length was 60 or 80 mm. With the exception of the flares, the PCSEMS was covered with a silicone membrane both inside and outside the metal mesh. In contrast, the FCSEMS was entirely covered, including the flares. PCSEMS, partially covered self‐expandable metal stent; FCSEMS, fully covered self‐expandable metal stent.

This multicenter, retrospective study of PCSEMSs was conducted in four referral centers in Japan (Kobe University Hospital, Akashi Medical Center, Kobe Medical Center, and Kita‐harima Medical Center). Patients with unresectable MDBO who underwent placement of PCSEMSs (Evolution Biliary Stent; Cook Ireland Ltd.; Figure 1a) in endoscopy units from September 2019 to July 2021 were included. The results were compared with those of a historical control of 42 consecutive patients who underwent placement of FCSEMSs (Evolution Biliary Stent; Cook Ireland Ltd.; Figure 1b) at four referral centers from January 2018 to August 2019.^10^ PCSEMSs and FCSEMSs used in this study were composed of a nitinol‐based braided cross‐wire structure with a platinum core, featuring proximal and distal flares designed to mitigate stent migration. The stent's outer diameter measured 10 mm, with a flare outer diameter of 11 mm and a flare length of 5 mm. The stents were available in lengths of either 60 or 80 mm, as illustrated in Figure 1. The PCSEMS was covered with a silicone membrane on both the inner and outer surfaces of the metal mesh, except for the flares. In contrast, the FCSEMS was fully covered, including the flares.

### Eligibility criteria

The inclusion and exclusion criteria were selected to match patients with the historical controls. The inclusion criteria were as follows: (1) being 20 years or older at the time of stent placement, and (2) being diagnosed with unresectable MDBO based on pathological and/or radiological features. The exclusion criteria were as follows: (1) previous failure to control jaundice or cholangitis via biliary drainage, (2) having an Eastern Cooperative Oncology Group Performance Status (ECOG‐PS) of 4, (3) having a coexisting hilar biliary obstruction, and (4) having surgically altered anatomical structures (for example, Billroth I, Billroth II, or Roux‐en‐Y reconstruction).

### Procedures

Both stents were placed at the biliary stricture during endoscopic retrograde cholangiopancreatography. Endoscopic sphincterotomy was performed before SEMS insertion. The length of the SEMS was selected according to the length and location of the biliary stricture. The proximal end of the SEMS was placed 10 mm beyond the biliary stricture and the distal end was placed 10 mm into the duodenal lumen across the papilla.

### Outcomes and definitions

The primary outcome was stent migration, and the secondary outcomes were RBO, overall survival, time to RBO (TRBO), clinical success, stent‐related adverse events, and re‐intervention.

The terms used in this study were defined with reference to the TOKYO criteria 2014 for transpapillary biliary stenting.^11^ Stent migration was diagnosed when re‐intervention revealed complete or partial migration of the SEMS resulting in RBO. RBO was defined as either stent occlusion or stent migration. Stent occlusion was defined as elevated blood concentrations of liver enzymes compared with baseline values, accompanied by biliary dilatation upon diagnostic imaging or features suggestive of stent occlusion upon endoscopy. Stent occlusion could be caused by tumor overgrowth, ingrowth, sludge formation, and food impaction. Overall survival was defined as the period from SEMS placement to death or the last follow‐up. Living patients were treated as censored cases at the date of final follow‐up. The TRBO was defined as the period from SEMS placement to RBO. Clinical success was defined as a decrease in the serum bilirubin concentration to less than 1.3 mg/dL or a decrease of at least 50% within 14 days. Stent‐related adverse events were also reported according to the TOKYO criteria 2014.^11^

### Statistical analysis

Continuous variables were compared using the Wilcoxon rank‐sum test, and categorical variables were compared using the χ^2^ test or Fisher's exact test, as appropriate. Overall survival was evaluated using the Kaplan–Meier method and compared using the log‐rank test. The TRBO was estimated via competing risks analysis; that is, the TRBO was calculated as one minus the cumulative incident rate, considering death as a competing risk. TRBO curves were compared using Gray's test.^12^ Risk factor analysis for stent migration was performed using Fine‐Gray regression,^13^ treating death or stent occlusion without death as competing risks. Patients who were lost to follow‐up were censored at the time of the last follow‐up. Variables with a *p*‐value < 0.05 in the univariate analysis were included in a multivariable analysis. A *p*‐value < 0.05 was deemed to indicate statistical significance.

All statistical analyses were performed using EZR (version 1.64; Saitama Medical Center, Jichi Medical University).^14^ EZR is a graphical user interface for R (version 4.31; The R Foundation for Statistical Computing). More precisely, it is a modified version of R commander (version 2.9‐1) with the addition of statistical functions commonly used in biostatistics.

## RESULTS

### Patient characteristics

This study included 39 patients who underwent PCSEMS placement (PCSEMS group). Meanwhile, 42 patients registered in a previous study were included as historical controls (FCSEMS group).^10^ Table 1 summarizes the patient characteristics. The groups did not significantly differ in terms of patient age, sex, ECOG‐PS, cause of MDBO, clinical stage, duodenal stenosis, prior drainage, white blood cell and C‐reactive protein concentrations before SEMS placement, stricture length, or stent length. However, the proportion of patients who received anti‐cancer treatment after SEMS placement was significantly lower (*p* = 0.008) in the PCSEMS group (15/39, 38%) than in the FCSEMS group (29/42, 69%).

**TABLE 1:** Patient characteristics.

Characteristics	PCSEMS (*n* = 39)	FCSEMS (*n* = 42)	*p*‐value
Age, median (range), years	74 (21–96)	68.5 (44–90)	0.11
Sex, *n* (%)			0.82
Male	22 (56)	22 (52)	
Female	17 (44)	20 (48)	
ECOG‐PS, *n* (%)			0.07
0	8 (21)	13 (31)	
1	16 (41)	23 (55)	
2	9 (23)	5 (12)	
3	6 (15)	1 (2)	
Cause of MDBO, *n* (%)			0.83
Pancreatic cancer	31 (79)	31 (74)	
Biliary cancer	5 (13)	7 (17)	
Others	3 (8)	4 (10)	
Clinical stage (UICC 8th), *n* (%)			0.54
Stage IV	19 (48)	27 (64)	
Stage III	12 (31)	10 (24)	
Stage II	5 (13)	3 (7)	
Stage I	3 (8)	2 (5)	
Duodenal stenosis			0.56
Yes	8 (21)	6 (14)	
No	31 (79)	36 (86)	
Prior drainage, *n* (%)			0.14
Endoscopic plastic stent placement	28 (72)	34 (81)	
Endoscopic nasobiliary drainage	0 (0)	2 (5)	
None	11 (28)	6 (14)	
WBCs, median (range), ×10^9^/L	6 (1.8–12.48)	5.56 (1.56–12.74)	0.34
CRP, median (range), ×10^4^ ug/L	1.37 (0.04–14.96)	0.86 (0.01–11.60)	0.16
Stricture length, median (range), cm	2.5 (1–5)	3 (1–5)	0.67
Stent length, *n* (%)			0.12
6 cm	15 (38)	24 (57)	
8 cm	24 (62)	18 (43)	
Anti‐cancer treatment after procedure, *n* (%)			0.008^*^
Chemotherapy/radiotherapy	15 (38)	29 (69)	
None	24 (62)	13 (31)	

Abbreviations: CRP, C‐reactive protein.; ECOG‐PS, Eastern Cooperative Oncology Group Performance Status; FCSEMS, fully covered self‐expandable metal stent; MDBO, malignant distal biliary obstruction; PCSEMS, partially covered self‐expandable metal stent; UICC, Union for International Cancer Control; WBC, white blood cell.

* A *p*‐value less than 0.05 was considered statistically significant.

### Clinical success, stent migration, RBO, and adverse events

Table 2 summarizes the clinical success, stent migration, RBO, and stent‐related adverse event rates. Clinical success was achieved in all 39 (100%) and 41 (98%) patients in the PCSEMS and FCSEMS groups, respectively (*p* = 0.99).

**TABLE 2:** Clinical outcomes.

Clinical outcomes	PCSEMS (*n* = 39)	FCSEMS (*n* = 42)	*p*‐value
Clinical success, *n* (%)	39 (100)	41 (98)	0.99
Recurrent biliary obstruction, *n* (%)	8 (21)	18 (43)	0.036^*^
Stent migration	1 (3)	15 (36)	<0.001^*^
Distal migration	1 (3)	11 (26)	0.004^*^
Proximal migration	0 (0)	4 (10)	0.12
Stent occlusion	7 (18)	3 (7)	0.31
Tumor overgrowth	4 (10)	0 (0)	0.049^*^
Sludge	3 (8)	3 (7)	0.99
Stent‐related adverse events, *n* (%)	8^‡^ (21)	8^§^ (19)	0.79
Pancreatitis	4 (10)	2 (5)	0.42
Cholecystitis	2 (5)	4 (10)	0.68
Non‐occlusive cholangitis	2 (5)	4 (10)	0.68
Liver abscess	1 (3)	0 (0)	0.48

Abbreviations: FCSEMS, fully covered self‐expandable metal stent; PCSEMS, partially covered self‐expandable metal stent.

* A *p*‐value less than 0.05 was considered statistically significant.

^‡^ One patient had two adverse events.

^§^ One patient had three adverse events.

The stent migration rate was significantly lower in the PCSEMS group, occurring in 1/39 (3%) patients compared with 15/42 (36%) in the FCSEMS group (*P *< 0.001). The rate of stent occlusion due to tumor overgrowth was significantly higher (*p* = 0.049) in the PCSEMS group (4/39, 10%) than that in the FCSEMS group which was not found (0/42, 0%). No stent occlusion due to tumor ingrowth or food impaction occurred in either group. Moreover, the RBO rate was significantly lower in the PCSEMS group, occurring in 8/39 (21%) patients compared with 18/42 (43%) in the FCSEMS group (*p* = 0.036).

Stent‐related adverse events occurred in 8/39 (21%) and 8/42 (19%) patients in the PCSEMS and FCSEMS groups, respectively (*p* = 0.79). The frequency of individual types of adverse events, including pancreatitis and cholecystitis, also did not differ significantly between the groups. In the PCSEMS group, although two patients with cholecystitis did not recover via conservative treatment, one recovered via percutaneous transhepatic gallbladder drainage, and the other via endoscopic ultrasonography‐guided gallbladder drainage. In the FCSEMS group, two patients with cholecystitis recovered via percutaneous transhepatic gallbladder drainage. Two other patients with cholecystitis recovered via conservative treatment. All other adverse events were mild and managed conservatively in both groups.

### Patient overall survival and TRBO

**FIGURE 2:**
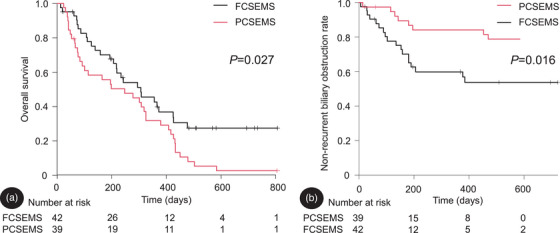
(a) Overall survival calculated using the Kaplan–Meier method. Overall survival was significantly shorter in the PCSEMS group (median: 247 vs. 307 days; log‐rank test, *p* = 0.027). (b) TRBO is estimated via competing risk analysis, with death treated as a competing risk. The non‐RBO rate at 6 months was 90% and 68% in the PCSEMS and FCSEMS groups, respectively. The TRBO was significantly longer in the PCSEMS group (Gray's test, *p* = 0.016). PCSEMS, partially covered self‐expandable metal stent; FCSEMS, fully covered self‐expandable metal stent; RBO, recurrent biliary obstruction; TRBO, time to RBO.

Figure 2a illustrates the overall survival. The median overall survival was 247 and 307 days in the PCSEMS and FCSEMS groups, respectively. Overall survival was significantly shorter in the PCSEMS group (log‐rank test, *p* = 0.027).

Figure 2b illustrates the TRBO. The non‐RBO rate at 6 months was 90% and 68% in the PCSEMS and FCSEMS groups, respectively. The TRBO was significantly longer in the PCSEMS group (Gray's test, *p* = 0.016).

### Risk factors for stent migration

Table 3 summarizes the univariate and multivariable analyses of the risk factors for stent migration. The univariate analysis revealed that PCSEMS placement was significantly associated with a lower risk of stent migration (subdistribution hazard ratio [SHR] = 0.056; 95% confidence interval [CI] = 0.01–0.42; *p* = 0.005) and that anti‐cancer treatment after procedure was significantly associated with a higher risk of stent migration (SHR = 6.57; 95% CI = 1.49–28.96; *p* = 0.013). With these two variables included in the multivariable analysis, only PCSEMS placement was significantly associated with a lower risk of stent migration (SHR = 0.077; 95% CI 0.01–0.60; *p* = 0.014).

**TABLE 3:** Risk factor analysis for stent migration.

	Univariate (Fine–Gray regression)		Multivariable (Fine‐Gray regression)	
Variable	SHR (95% CI)	*p*‐Value	SHR (95% CI)	*p*‐Value
Pancreatic cancer	0.94 (0.31–2.88)	0.91		
Duodenal stenosis	1.07 (0.31–3.74)	0.91		
Prior biliary drainage	0.67 (0.22–2.03)	0.47		
Stricture length	0.93 (0.53–1.61)	0.79		
Stent length: 80 mm	0.72 (0.44–1.19)	0.2		
Anti‐cancer treatment after procedure	6.57 (1.49–28.96)	0.013^*^	3.97 (0.87–18.24)	0.076
PCSEMS	0.056 (0.01–0.42)	0.005^*^	0.077 (0.01–0.60)	0.014^*^

Fine‐Gray regression; competing event: death or stent occlusion without death.

Abbreviations: CI, confidence interval; PCSEMS, partially covered self‐expandable metal stents; SHR, subdistribution hazard ratio.

* A *p*‐value less than 0.05 was considered statistically significant.

### Re‐intervention

Table 4 summarizes the re‐interventions. In both groups, the reason for re‐intervention was RBO in all cases. In the PCSEMS group, re‐intervention was attempted in seven of eight RBO cases (88%); however, it was not performed in one case due to poor general condition resulting from advanced pancreatic cancer. In the FCSEMS group, re‐intervention was attempted in all 18 RBO cases (100%). The rate of re‐intervention attempts was significantly lower in the PCSEMS group, with 7/39 (18%) compared with 18/42 (43%) in the FCSEMS group (*p* = 0.029). Successful rates of re‐intervention did not significantly differ (*p* = 0.28) between the PCSEMS group (6/7, 86%) and the FCSEMS group (18/18, 100%). In the PCSEMS group, re‐intervention was unsuccessful in one case because the endoscope could not pass through the pyloric stenosis caused by cancer dissemination. SEMS removal was successful in 2/2 PCSEMS cases and 6/6 FCSEMS cases, with a 100% success rate in both groups. The methods of re‐intervention in the PCSEMS and FCSEMS groups were as follows (PCSEMS vs. FCSEMS): stent placement after SEMS removal, 2/6 (33%) versus 6/18 (33%; *p* = 0.99); additional stent placement in a stent‐in‐stent manner, 2/6 (33%) versus 0/18 (0%; *p* = 0.054); stent placement following complete distal SEMS migration 0/6 (0%) versus 11/18 (61%; *p* = 0.016); and sludge sweeping using a balloon catheter 2/6 (33%) versus 1/18 (6%; *p* = 0.14). No patients underwent percutaneous transhepatic biliary drainage or endoscopic ultrasonography‐guided biliary drainage as a re‐intervention. The median time to re‐intervention was 143 days (range, 14–469 days) in the PCSEMS group and 120 days (range, 6–385 days) in the FCSEMS group. The detail of re‐interventions in the PCSEMS group is summarized in Table 5.

**TABLE 4:** Re‐interventions.

Re‐interventions	PCSEMS (*n* = 39)	FCSEMS (*n* = 42)	*p*‐value
Attempts at re‐intervention, *n* (%)	7/39 (18)	18/42 (43)	0.029^*^
Success of re‐intervention, *n* (%)	6/7 (86)^‡^	18/18 (100)	0.28
Attempts at SEMS removal, *n* (%)	2/7 (29)	6/18 (33)	0.99
Success of SEMS removal, *n* (%)	2/2 (100)	6/6 (100)	0.99
Methods of re‐intervention			
Stent placement after SEMS removal, *n* (%)	2/6 (33)	6/18 (33)	0.99
Stent placement in a stent‐in‐stent manner, *n* (%)	2/6 (33)	0/6 (0)	0.054
Stent placement following complete distal SEMS migration, *n* (%)	0/6 (0)	11/18 (61)	0.016^*^
Sludge sweeping using a balloon catheter, *n* (%)	2/6 (33)	1/18 (6)	0.14
Time to re‐intervention, median (range), days	143		
(14–469)	120		
(6–385)	0.3		

Abbreviations: FCSEMS, fully covered self‐expandable metal stent; PCSEMS, partially covered self‐expandable metal stent; RBO, recurrent biliary obstruction; SEMS, self‐expandable self‐expanding metal stent.

* A *p*‐value less than 0.05 was considered statistically significant.

‡ Re‐intervention was unsuccessful in only one patient because the endoscope could not pass through the pyloric stenosis.

**TABLE 5:** Detail of re‐interventions in the partially covered self‐expandable metal stent group.

Case	MDBO	Chemotherapy	RBO	TRBO (days)	Treatment details during re‐intervention
1	PC	GnP	Overgrowth	196	Placement of a new PCSEMS after removal of the PCSEMS
2	PC	None	Overgrowth	130	Placement of a new UCSEMS after removal of the PCSEMS
3	PC	None	Overgrowth	469	Placement of an additional PS inside the PCSEMS
4	PC	GnP	Overgrowth	143	Placement of an additional PCSEMS inside the PCSEMS
5	PC	None	Sludge	116	Sludge sweeping using a balloon catheter
6	PC	GnP	Sludge	14	Sludge sweeping using a balloon catheter
7	PC	GnP	Sludge	453	Unsuccessful because of pyloric stenosis by cancer dissemination

Abbreviations: GnP, gemcitabine plus nab‐paclitaxel; MDBO, malignant distal biliary obstruction; PC, pancreatic cancer; PCSEMS, partially covered self‐expandable metal stent; PS, plastic stent; RBO, recurrent biliary obstruction; TRBO, time to RBO; UCSEMS, uncovered self‐expandable metal stent.

## DISCUSSION

This multicenter, retrospective study was conducted to investigate the clinical outcome of PCSEMSs in comparison with a historical control of patients receiving FCSEMSs, specifically in terms of their anti‐migration effects. Although the rate of stent occlusion due to tumor overgrowth was significantly higher in the PCSEMS group (10% vs. 0%; *p* = 0.049), the rates of stent migration (3% vs. 36%; *p *< 0.001) and RBO (21% vs. 43%; *p* = 0.036) were significantly lower in the PCSEMS group. Moreover, the TRBO was significantly longer in the PCSEMS group (Gray's test, *p* = 0.016).

The main disadvantage of FCSEMSs is that they are prone to migrate because they are not embedded in the bile duct.^15^, ^16^, ^17^, ^18^ Structural attributes of SEMSs that contribute to stent migration include mechanical properties such as the axial force (AF) and radial force (RF), the anti‐migration system, and the membrane materials covering the stents. In fact, rates of FCSEMS migration vary from 4% to 36% among stent manufacturers and reports.^8^, ^9^, ^10^, ^19^, ^20^, ^21^, ^22^, ^23^ The AF is the straightening force on the central axis of the SEMS and contributes to its flexibility with respect to the bile duct.^24^ Isayama et al. speculated that a high AF may cause the SEMS to not fit well in the bile duct and migrate.^25^ The RF is the expanding force against the stricture that contributes to its dilation.^24^ Nakai et al. reported that a low RF was a risk factor for stent migration.^23^ A previous study in which the AF and RF were compared among stent types revealed that the type of PCSEMSs and FCSEMSs that we evaluated in this study both have a high AF and moderate RF.^24^ These mechanical properties might have contributed to the high rate of FCSEMS migration in this study.

To overcome stent migration, various anti‐migration systems have been developed, including flared ends,^9^, ^10^ dumbbell‐shaped flared ends,^22^ anchoring fins,^26^ and raised bands on the stent body.^21^ Minaga et al. reported that the prevention of migration in braided‐type FCSEMSs depended on the outer diameter, length, and shape of the flared ends. They demonstrated that larger flared ends (both in diameter and length) and a larger angle between the stent body and the flare helped prevent stent migration. The FCSEMSs used in this study had smaller flared ends with a smaller angle between the stent body and the flared ends. These features of the flared ends could be a possible reason for the high migration rate of the FCSEMSs used in this study. Meanwhile, the PCSEMSs used in this study have “uncovered” flared ends, which appear to be effective in preventing stent migration.

To our knowledge, the clinical performance of PCSEMSs and FCSEMSs in patients with MDBO has been compared in two studies: a retrospective study by Yokota et al. and a prospective study by Kogure et al. In both studies, the same SEMSs (WallFlex Biliary RX Stent; Boston Scientific Corp.) were used.^8^, ^9^ In our study, the rate of stent‐related adverse events did not significantly differ between the PCSEMS and FCSEMS groups, which was consistent with those previous studies.^8^, ^9^ However, the results differed in other respects. For example, in our study and that by Yokota et al., the stent‐migration rate was significantly lower and TRBO was significantly longer with PCSEMSs,^8^ whereas Kogure et al. reported no significant differences.^9^ In addition, the RBO rate was significantly lower in the PCSEMS group only in our study. To the best of our knowledge, our study is the first to reveal a significant difference in the rate of RBO between FCSEMSs and PCSEMSs. Possible reasons for these discrepancies include differences in study design, the number of patients included, patient backgrounds, and stent manufacturers.

A recent meta‐analysis comparing the clinical outcomes of PCSEMSs and FCSEMSs showed negligible differences in the rate of RBO and adverse events, but the PCSEMS group had a lower stent‐migration rate, higher overgrowth rate, and longer TRBO.^28^ These results were generally consistent with our study. A major limitation of this meta‐analysis was that only a few studies directly comparing the clinical outcomes of PCSEMS and FCSEMS were included. Thus, further studies are needed to directly compare the performance of these SEMSs.

Removability is an important factor to consider when the performance of SEMSs is evaluated. In addition, the ease of removal of SEMSs is important in the case of stent‐related adverse events such as post‐endoscopic retrograde cholangiopancreatography cholecystitis and pancreatitis. The reported success rate of FCSEMS's removal is 91%‐100%.^9^, ^10^, ^15^ PCSEMSs may be more difficult to remove owing to the uncovered flared ends. The reported success rate for PCSEMS removal is 78%–96%.^29^, ^30^ In our study, SEMS removal was successful in two of two PCSEMS cases and six of six FCSEMS cases, a 100% success in both groups. For the two PCSEMS cases, the time from stent placement to removal was 130 and 196 days, respectively. Even PCSEMSs may be removable if the time from stent placement is short.

This study has several limitations. A major limitation is its retrospective nature and historical controls; therefore, the timing of stent placement differed between the two groups. Furthermore, the samples were small. Another limitation is that the patient backgrounds, especially the rate of anti‐cancer therapy and overall survival, were not matched. However, Fine‐Gray regression did not identify any independent predictors of stent migration other than the type of SEMSs. The strengths of this study lie in its multicenter design and the use of different types of stents compared to previous studies.

In conclusion, this study suggests that the anti‐migration effect of PCSEMSs reduces the risk of RBO and prolongs the TRBO.

## CONFLICT OF INTEREST STATEMENT

None.

## ETHICS STATEMENT

This study was collectively reviewed and approved by the ethics committee of the Kobe University Hospital (No. B220193) and conducted in accordance with the principles of the Declaration of Helsinki. Because of the retrospective study with the historical control, the requirement for informed consent was waived. Study information was then published on our website and eligible patients, including those with FCSEMS, were offered the opportunity to opt‐out. Clinical trial registration was not applicable. Animal studies were not applicable.
